# Banding Karyotyping From Peripheral Blood Is a Useful Method for the Diagnostic Workup of Myelodysplastic Neoplasia and CMML

**DOI:** 10.1002/gcc.70177

**Published:** 2026-10-09

**Authors:** Cornelia Heusler, Barbara Hildebrandt, Felicitas Schulz, Kathrin Nachtkamp, Corinna Strupp, Valerie Flatten, Sascha Dietrich, Norbert Gattermann, Ulrich Germing

**Affiliations:** ^1^ Department of Hematology, Oncology and Clinical Immunology Heinrich‐Heine University Duesseldorf Germany; ^2^ Institute of Human Genetics, Heinrich‐Heine University Duesseldorf Germany

**Keywords:** IPSSR, karyotype risk groups, karyotyping peripheral blood, myelodysplastic syndromes, prognosis

## Abstract

This retrospective study involving 155 patients from the Düsseldorf MDS Registry compared cytogenetic banding from peripheral blood (PB) and bone marrow (BM) in patients with myelodysplastic syndromes (MDS) and chronic myelomonocytic leukemia (CMML). For 63 of these patients, paired PB and BM cytogenetic analyses were available that were conducted no more than 3 months apart. Concordant ISCN results were found in 81%, with no significant differences in the total number of metaphases or aberrant metaphases between PB and BM. Furthermore, IPSS‐R/‐M scorings, derived from PB and BM, turned out to be comparable, with only 7 out of 63 patients classified into a different cytogenetic risk category. This implies that aberrant clones are not underrepresented in PB and that cytogenetic findings from PB can reliably reflect those obtained from BM.

## 1 Introduction

Myelodysplastic syndrome (MDS) is one of the more common bone marrow (BM) diseases with an incidence of 4 in 100 000 per year, rising up to 30 in 100 000 per year for the population aged 70 and above [1]. Due to dysplastic, ineffective blood cell production, MDS patients show frequent cytopenias and increased risk of progression to acute myeloid leukemia (AML) [1]. During the course of the disease, genetic instability cause cytogenetic heterogeneity leading to karyotype evolution and the emergence of subclones [2]. Despite the development of novel genetic diagnostics, conventional chromosome banding (CCB) is an integral part of the classification and characterization of MDS [3]. Chromosomal abnormalities, which have a significant influence on prognosis as well as treatment decisions, are essential to monitor treatment response [4, 5]. Clonal chromosomal aberrations are detected in around 50% of patients with primary MDS and in approximately 80% of patients with post cytotoxic MDS [3]. Most common aberrations include −5/5q−, −7/7q−, +8, −18/18q−, 20q−, −Y, +21, −17/17p−, inv(3)/t(3q), −13/13q− and many more, highlighting the profound heterogeneity of the disease [6].

Cytogenetic aberrations are part of the revised international prognostic scoring system (IPSS‐R), developed in 2012, which separates patients into five risk categories according to cytogenetics, BM blast percentage, hemoglobin, platelets, and absolute neutrophil count [4]. A novel tool for risk stratification was developed in 2022 and called the molecular international prognostic scoring system (IPSS‐M). Here, patients are assigned to a six‐category risk schema, considering hematologic parameters, cytogenetic abnormalities, and somatic mutations of 31 genes [7]. In general, complex karyotypes and clonal evolution are associated with an unfavorable prognosis, whereas normal karyotypes as well as isolated 5q− or −Y are associated with a good prognosis. In clinical practice, BM aspirates are the gold standard to acquire adequate material for metaphase evaluation by CCB. However, BM biopsies can induce procedure‐related pain, as well as emotional distress and anxiety [8]. This puts an additional burden on an elderly and often frail patient population and may lead to patient refusal. Furthermore, BM aspirations may not be possible due to dry tap.

Cytogenetic studies can be extended to the use of fluorescence in situ hybridization (FISH), allowing chromosomal analyses in nonproliferating interphase nuclei. In addition, microarray analyses and next‐generation sequencing are important for the identification of molecular genetic mutations. Nonetheless, CCB is an inexpensive and time‐tested way to detect both numerical and structural chromosomal abnormalities and monitor their clonal evolution [9].

Generally, it is more difficult to perform CCB from peripheral blood (PB) in patients with MDS, due to scarcity of cells capable of performing mitosis. To date, FISH analysis of enriched circulating CD34+ cells is the only established method for cytogenetic analysis from PB [10]. Circulating CD34+ cells are representative of the clonal population in the BM and allow frequent monitoring during therapy. If CCB could be done using PB cells not enriched for CD34+ cells, with FISH providing complementary data, the need for invasive BM biopsies could be drastically reduced. Therefore, we evaluated CCB of PB cells regarding the detectability of clonal aberrations identified in aspirated BM cells.

## 2 Patients and Methods

### 2.1 Patient Cohort

Overall, the Düsseldorf MDS registry contained more than 9300 patients diagnosed with MDS as of December 2022. Of these, 3189 patients with an initial diagnosis between 1982 and 2022 had available karyotype information and therefore were included in the study database. The diagnosis and classification of MDS were performed according to the WHO 2022 classification of myeloid neoplasms [11]. Patient data were compiled in Düsseldorf, one of the collaborating centres in Germany. The Düsseldorf MDS registry collects basic demographic and clinical data including cytological and histological findings from BM aspirates obtained at diagnosis and during follow‐up, as well as cytogenetic and molecular genetic results. Upon registration, a unique identification number is assigned to each patient, ensuring pseudonymization throughout all subsequent analyses. To ensure compliance with the latest disease classifications and diagnostic criteria, the MDS registry is regularly updated. The main purpose of this analysis was to compare the quality of cytogenetic banding from BM and PB in MDS patients. Therefore, only patients were included whose BM and PB cytogenetic analyses were conducted no more than 3 months apart.

### 2.2 Cytogenetic Analysis

Cytogenetic data were retrieved from the MDS registry. Karyotypes were documented according to ISCN nomenclature (version 2020), indicating chromosome number, sex chromosomes, chromosomal abnormalities, and the number of metaphases analyzed [12]. The IPSS karyotype scores, including the number of clonal abnormalities, were generated according to international guidelines.

For cultivation, BM (5–10 mL) or PB samples (3–10 mL) were collected in heparin tubes. Upon arrival in the Institute of Human Genetics, University Hospital Düsseldorf, samples were washed twice with Roswell Park Memorial Institute (RPMI) medium. Cell counts were determined and adjusted for optimal cell density. For each patient, three parallel cultures were prepared using the culture media Panserin and MarrowMAX, with Panserin applied in two cultures with different incubation times. Cultures were incubated for 24, 48, or up to 72 h. Hereafter, cells were treated with Colcemid for 6–14 h, arresting cells in metaphase by preventing the formation of a spindle apparatus. By varying different culture media, incubation times, and Colcemid exposure durations, the percentage of culture failures was minimized. In preparation for fixation, hypotonic treatment with potassium chloride was performed to increase cell volume, followed by erythrocyte lysis using Ibraimov solution (5% acetic acid) to prevent formation of gelatinous cell aggregates. Cells were fixed in a three‐step fixation procedure using a methanol/acetic acid solution (3:1). The acetic acid stabilizes the cell walls, while the methanol lowers water content, allowing long‐term storage for years. After fixation, cell suspensions were applied to microscope slides and aged at 60°C for 16 h. Chromosome banding was performed using standard trypsin digestion followed by Giemsa staining (G‐banding). Finally, preparations were permanently mounted with Entellan to preserve the stained chromosomes for long‐term analysis. Depending on sample quality, up to 16 slides per patient were prepared to reduce the overall dropout rate. Cultures were established without mitogenic stimulation, as cytogenetic analysis in MDS can be performed on spontaneously proliferating malignant hematopoietic progenitor cells [13]. No samples were excluded from cytogenetic analysis based on sample quality. Karyotypes were reported as having limited evaluability when the chromosome banding resolution was ≤ 250 bands or when fewer than 10 evaluable metaphases were available for analysis.

Metaphases were captured using an automated Metafer MSearch Metaphase Finder (Metasystems, Altlussheim, Germany). The captured metaphases were randomly selected for complete G‐banding analysis (band to band). As part of the routine procedure, a karyotyping protocol was prepared to document all anomalies observed in each metaphase. Metaphases were numbered according to the order in which they were analyzed.

### 2.3 Statistical Analysis

For statistical analysis, the program Statistical Package for the Social Sciences (SPSS; IBM, Armonk, NY, USA), version 31.0.1.0 for Windows, was used. Number of metaphases and aberrant metaphases in BM and PB were presented as mean ± standard deviation (SD). Paired comparisons between BM and PB were performed using two‐tailed paired samples *t*‐tests, and corresponding 95% confidence intervals (CI) were calculated. Cytogenetic risk categories according to IPSS‐R/‐M from BM and PB were analyzed as paired ordinal variables by the Wilcoxon signed‐rank test.

### 2.4 Ethical Considerations

This study was performed in line with the principles of the Declaration of Helsinki. Approval was granted by the Ethics Committee of the Heinrich‐Heine University Duesseldorf (March 8, 2023/No. 3973).

## 3 Results

Three thousand one hundred and eighty nine patients from the Düsseldorf MDS Registry with an initial diagnosis between 1982 and 2022 underwent BM cytogenetic analysis. An abnormal karyotype was identified in 1646 patients (51.6%). In 118 additional patients cytogenetic analysis was performed both in BM and PB samples, with PB sampling being performed once or multiple times. For 18 patients, PB was tested exclusively without available BM cytogenetic analyses. In total, PB cytogenetic data from 155 patients was analyzed, of whom 69 (4.5%) demonstrated an abnormal karyotype (Table 1).

**TABLE 1:** Karyotypes analyzed (number of samples examined).

	Metaphases analyzed (BM)	Metaphases analyzed (PB)
Frequency (*n*)	3189	155
Abnormal karyotype	1646	69
Median number of metaphases evaluated per sample	20	24
Minimum number of metaphases evaluated per sample	1	2
Maximum number of metaphases evaluated per sample	120	50

Among those 155 patients, 63 had cytogenetic analyses of BM and PB less than 3 months apart. Within the 63 matches, patient characteristics were analyzed according to blast count in PB and BM and WHO 2022 classification. Blast count categories were based on blast percentage thresholds defined in the WHO 2022 classification (Table 2). For analysis of WHO 2022 subgroups, cases were classed as low blast MDS (SLD, MLD, and hypocellular MDS), increased blasts MDS (IB1, IB2, fibrosis), or genetically defined MDS (SF3B1 mutation and isolated del(5q)) (Table 3). Out of 63 matched patients, 45 could be assigned to a WHO 2022 category. Blast count data from both PB and BM were available for all 45 of these patients. For the remaining 18 patients, no information on either WHO 2022 classification nor blast count data was available in the MDS registry. Among the 45 patients with available blast count data, 39 (86.7%) had a PB blast count of < 2%; meanwhile, 31 (68.9%) had a BM blast count of < 10%.

**TABLE 2:** Patient characteristics: Blast count.

Blast count in PB	Frequency in PB, *n* (%)	Blast count in BM	Frequency in BM, *n* (%)
< 2%	39 (61.9%)	< 5%	18 (28.6%)
		5%–9%	13 (20.6%)
2%–4%	3 (4.8%)	10%–19%	10 (15.9%)
5%–19%	3 (4.8%)	≥ 20%	4 (6.3%)
Missing data	18 (28.6%)	Missing data	18 (28.6%)
Total number	63 (100%)	Total number	63 (100%)

**TABLE 3:** Patient characteristics: WHO 2022 MDS and MDS/MPN subtypes.

MDS subtype (according to WHO 2022)	Frequency, *n* (%)
Low blast count (SLD, MLD, hypocellular MDS)	11 (17.5%)
Genetically defined MDS (del(5q), SF3B1)	6 (9.5%)
Increased blast count (IB1, IB2, fibrosis)	15 (23.8%)
CMML 1	6 (9.5%)
CMML 2	3 (4.8%)
AML MDC (former RAEB‐T)	4 (6.3%)
Missing data	18 (28.6%)
Total	63 (100%)

Table 4 summarizes the frequencies of ISCN findings in BM and PB samples. In 51 paired samples (81%), BM and PB analysis showed identical karyotype according to ISCN in both BM and PB samples, whereas 12 pairs (19%) demonstrated discrepant findings. Among the 51 identical cases, only 9 pairs (17.6%) showed matching numbers of analyzed metaphases, while 42 pairs had varying numbers (82.4%). In contrast, 41 concordant pairs (80.4%) demonstrated a match in the number of aberrant metaphases, with 10 pairs (19.6%) showing no match.

**TABLE 4:** Frequencies of ISCN findings according to WHO in BM versus PB.

	Frequency, *n* (%)	Match: number of metaphases, *n* (%)	Match: number of aberrant metaphases, *n* (%)
BM = PB according to ISCN	51 (81%)	Yes: 9 (17.6%)	Yes: 41 (80.4%)
		No: 42 (82.4%)	No: 10 (19.6%)
BM ≠ PB according to ISCN	12 (19%)	Yes: 4 (33.3%)	Yes: 0 (0%)
		No: 8 (66.7%)	No: 12 (100%)
No time correlation	92		
Total number of analyses in PB	155		

Number of metaphases were compared in patients with matching cytogenetic aberrations in BM and PB. Metaphase abundance was analyzed using two clinically relevant thresholds: ≥ 10 metaphases representing routine threshold commonly applied in diagnostic cytogenetics (Table 5), and ≥ 20 metaphases, corresponding to the International System for Human Cytogenetic Nomenclature (ISCN) recommendations (Table 6) [2]. In PB, the threshold of 10 was surpassed in 65.1%, and in BM analysis in 66.7%. Regarding aberrant metaphases, the threshold of 10 was surpassed in 19% of PB and 12.7% of BM samples. When applying the ISCN threshold, 47.6% of PB and 55.6% of BM samples obtained ≥ 20 metaphases. The proportion of samples with ≥ 20 aberrant metaphases was 7.9% in both PB and BM.

**TABLE 5:** Abundancy of metaphases and aberrant metaphases in BM versus PB (threshold ≥ 10).

	Frequency, *n* (%)	Absolute number of metaphases in PB ≥ 10	Absolute number of metaphases in BM ≥ 10	Absolute number of aberrant metaphases in PB ≥ 10	Absolute number of aberrant metaphases in BM ≥ 10
BM = PB according to ISCN	51 (81%)	Yes: 41 (65.1%)	Yes: 42 (66.7%)	Yes: 12 (19%)	Yes: 8 (12.7%)
		No: 10 (15.9%)	No: 9 (14.3%)	No: 39 (61.9%)	No: 43 (68.3%)
BM ≠ PB according to ISCN	12 (19%)	Yes: 9 (14.3%)	Yes: 10 (15.9%)	Yes: 4 (6.3%)	Yes: 6 (9.5%)
		No: 3 (4.8%)	No: 2 (3.2%)	No: 8 (12.7%)	No: 6 (9.5%)
No time correlation	92				
Total number of analyses in PB	155	Yes: 136 (87.7%)		Yes: 54 (34.8%)	
		No: 19 (12.3%)		No: 101 (65.2%)	

**TABLE 6:** Abundancy of metaphases and aberrant metaphases in BM versus PB (threshold ≥ 20).

	Frequency, *n* (%)	Absolute number of metaphases in PB ≥ 20	Absolute number of metaphases in BM ≥ 20	Absolute number of aberrant metaphases in PB ≥ 20	Absolute number of aberrant metaphases in BM ≥ 20
BM = PB according to ISCN	51 (81%)	Yes: 30 (47.6%)	Yes: 35 (55.6%)	Yes: 5 (7.9%)	Yes: 5 (7.9%)
		No: 21 (33.3%)	No: 16 (25.4%)	No: 46 (73.0%)	No: 46 (73.0%)
BM ≠ PB according to ISCN	12 (19%)	Yes: 7 (11.1%)	Yes: 9 (14.3%)	Yes: 3 (4.8%)	Yes: 5 (7.9%)
		No: 5 (7.9%)	No: 3 (4.7%)	No: 9 (14.3%)	No: 7 (11.1%)
No time correlation	92				
Total number of analyses in PB	155	Yes: 110 (70.1%)		Yes: 31 (19.7%)	
		No: 47 (29.9%)		No: 124 (79.0%)	

A paired samples *t*‐test was conducted to compare the number of metaphases in PB versus BM as well as the number of aberrant metaphases in PB versus BM (Table 7). Metaphase counts were slightly higher in BM (*M* = 19.40; SD = 7350) than in PB (*M* = 18.25; SD = 8679). A paired samples *t*‐test showed that the number of metaphases did not differ significantly between PB and BM (t(62) = 0.944; *p* = 0.349 [two‐tailed]; 95% CI [−1.277, 3.563]).

**TABLE 7:** Paired comparison of metaphase and aberrant metaphase counts between bone marrow and peripheral blood.

Comparison	Mean difference	SD of difference	t(62)	*p* (two‐tailed)	95% CI (lower, upper)
Metaphases (BM/PB)	1.143	9.608	0.944	0.349	−1.277, 3.563
Aberrant metaphases (BM/PB)	0.937	8.580	0.866	0.390	−1.224, 3.097

Regarding aberrant metaphases, counts were again slightly higher in BM (*M* = 5.59; SD = 9012) than in PB samples (*M* = 4.65; SD = 8024). Aberrant metaphase count also did not significantly differ between BM and PB (t(62) = 0.866; *p* = 0.390 [two‐tailed]; 95% CI [−1.224, 3.097]). Among the 63 temporally correlated pairs, 41 cases (65.1%) demonstrated identical numbers of aberrant metaphases. In 11 cases (17.5%), a higher number of aberrant metaphases was observed in PB compared with BM, whereas in another 11 cases (17.5%), the number of aberrant metaphases was greater in BM than in PB.

Finally, risk stratification according to IPSS‐R and IPSS‐M was evaluated. Patients were assigned to risk groups between 1 (*very good*) and 5 (*very poor*), based on the cytogenetic risk category defined in both scoring systems [4, 7]. Since none of the patients was assigned into the “very poor” category, the effective range in this cohort was between 1 (*very good*) and 4 (*poor*). Descriptive statistics showed that mean IPSS‐R/‐M scores were comparable between BM and PB assessments (Table 8). For the BM‐based IPSS‐R/‐M, the mean score was 2.35 (SD = 0.79; range 1–4), while the PB‐based IPSS‐R/‐M showed a mean of 2.33 (SD = 0.78; range 1–4).

**TABLE 8:** Comparison of IPSS‐R/‐M cytogenetic categories between bone marrow and peripheral blood using the Wilcoxon signed‐rank test.

Measure	*N*	Mean ± SD	Range	Negative ranks (PB < BM)	Positive ranks (PB > BM)	Ties (PB = BM)	*Z*
IPSS‐R/IPSS‐M	63	2.35 ± 0.79 (BM) 2.33 ± 0.78 (PB)	1–4	4	3	56	−0.345

A Wilcoxon signed‐rank test was conducted to compare paired IPSS‐R/‐M values derived from BM and PB. The analysis identified four negative ranks (PB <BM), indicating that with these patients the cytogenetic risk score derived from PB was better than that from BM, resulting in four patients being classified into a lower risk category.

Conversely, three positive ranks (PB > BM) reflected higher risk scores in PB compared with BM, leading to three patients being classified into a poorer risk category based on PB.

Most cases (*n* = 56) showed no difference between the two measures. The test indicated no statistically significant difference between IPSS‐R/‐M scores obtained from PB and BM (*Z* = −0.35, *p* = 0.73).

## 4 Discussion

At the time of initial diagnosis of myelodysplastic neoplasms, cytogenetic analysis is mandatory for accurate disease classification and prognostic stratification. Currently, invasive BM biopsy procedures are necessary to determine cytogenetic risk and histomorphology for reliable assessment. Cytogenetic analysis is performed using CCB and may be complemented by FISH.

This study addressed whether CCB from PB can reliably detect clonal aberrations observed in BM. In 63 temporally matched sample pairs, we found a high concordance rate of 81% (51 of 63 pairs) regarding chromosomal findings. These results are in line with an earlier study on MDS patients by Cherry and colleagues, who observed a match in 83.5% (43 of 46 pairs) between BM and PB [14]. However, they found CCB to be unsuccessful in 54%. In their analysis, discordances were generally attributed to a low tumor burden in the PB of MDS. In addition, poor quality of metaphases may have led to chromosomal aberrations being interpreted incorrectly or remaining undetected [9]. In the Institute of Human Genetics, University Hospital Düsseldorf, the culture failure rate for CCB from PB is below 1%. This is likely due to the use of three culture media in parallel, increasing the probability of adequate cell proliferation. Although this approach uses more resources, it reduces the risk of complete culture failure compared with single‐ or dual‐medium protocols. Furthermore, metaphase screening is conducted on up to 16 slides per sample, enhancing detection sensitivity. Other laboratories often limit analysis to three to four slides, which may prematurely classify low proliferative samples as culture failures. Finally, prolonged exposure to Colcemid, as routinely performed in our laboratory, appears to increase the success rate, particularly in samples with low cell counts [15].

Because CCB from PB can be technically challenging, obtaining an adequate number of metaphases is essential for reliable clonal detection. According to Steidl and colleagues, there is a statistically significant difference in the detection of cell clones in a patient group with ≤ 19 metaphases analyzed compared with a patient group with ≥ 20 metaphases analyzed [2]. If an abnormal clone is detected, the analysis can be limited to 10 metaphases [9]. At University Hospital Düsseldorf metaphase evaluation is routinely continued beyond this threshold, because clone size itself provides prognostic information on overall survival (OS) and risk of AML evolution [16]. In our study, among the 51 samples with matching ISCN results, the proportion of samples with ≥ 10 aberrant metaphases was higher in PB than BM (19% [12 out of 51 samples] vs. 12.7% [8 out of 51 samples]). No difference was observed regarding the number of samples with ≥ 20 aberrant metaphases, with both PB and BM showing a percentage of 7.9% (5 out of 51 samples). Regarding all 63 time‐paired samples, the number of samples with ≥ 10 aberrant metaphases was similar between PB (16 out of 63) versus compared with BM (14 out of 63), while the number of samples with ≥ 20 aberrant metaphases was only slightly higher in BM (10 out of 63) compared with PB (8 out of 63).

Although it may be suggested that high blast counts, as typically observed in higher risk MDS, are necessary for successful CCB, this was not accurate for this cohort. Among patients with available blast count data, 68.9% had < 10% blasts in BM and 86.7% had < 2% blasts in PB. Additionally, the majority of patients were categorized in a more favorable WHO subtype. These findings argue against systematic underrepresentation of aberrant clones in PB due to lower circulating tumor burden, as presumed by Cherry and colleagues [14]. However, if the proportion of malignant cells is indeed lower in PB than in BM, the resulting quantitative disadvantage may be compensated by a higher proliferative capacity of malignant PB cells in culture compared with nonmalignant PB cells.

In addition, in the 51 samples with matching ISCN, the number of samples with aberrant metaphases—including those with counts below 20—matched between BM and PB in 80.4%. Although the total number of aberrant metaphases was slightly higher in BM (*M* = 5.59) than in PB (*M* = 4.65), the number of samples with high aberrant metaphases was identical in PB and BM. This leads us to the conclusion that, under optimized culture conditions, a systematic loss of sensitivity for detection of aberrant clones does not occur.

From a clinical perspective, the most relevant finding of this analysis is consistency of cytogenetic risk stratification. After applying cytogenetic IPSS‐R/‐M scores, only 7 out of 63 patients experienced a shift in risk stratification, with four patients being classified into a higher risk category in PB and three patients being classified into a lower risk category based on PB. Since IPSS‐R/‐M scores are strongly influenced by cytogenetic risk parameters and highly relevant for therapeutic decisions, the observed consistency between cytogenetic results from PB and BM is reassuring.

Another important clinical aspect of cytogenetic testing is longitudinal monitoring of clonal evolution, which refers to the emergence of new genetic aberrations [17, 18, 19, 20]. This monitoring is particularly relevant because disease progression in MDS is driven by ongoing genomic changes, including sequential acquisition of novel genetic aberrations and clonal expansion [18]. While these processes occur more frequently in higher risk MDS, they are also observed in lower risk groups with a reported rate of one clonal event every 4 years [18]. In a separate cohort of 549 MDS patients, clonal evolution was detected in 24% of cases [19]. A clear adverse effect of clonal evolution on clinical progression to AML and OS could be found [19]. Similarly, Schanz and colleagues observed clonal evolution of 729 patients with MDS and demonstrated that patients who developed clonal evolution, again had a significantly shorter median OS than those without evidence of clonal evolution (18.0 vs. 53.9 months) [17].

Together, these findings highlight the prognostic significance of repeated cytogenetic assessment and emphasize the importance of detecting clonal evolution, as they provide relevant prognostic information and may influence prognostic assessment and therapeutic decision‐making [17, 18, 19]. Less invasive techniques could simplify longitudinal monitoring and improve the timely detection of clinically relevant new clones.

Braulke and colleagues used sequential FISH analyses of immunomagnetically enriched circulating CD34+ PB cells for therapy monitoring in MDS patients [10]. CD34+ PB cells were shown to carry the same chromosomal aberrations as BM cells [21]. This method works for the early detection of lenalidomide treatment failure in MDS patients [22] and for cytogenetic monitoring in patients treated with azacitidine [23]. Cytogenetic responders showed a significantly higher hematologic improvement rate and a significantly better OS than nonresponders [23]. Accordingly, early identification of responders and nonresponders is clinically important for treatment decisions. Our data show that, in addition to targeted FISH analysis, CCB from PB is feasible in a substantial proportion of patients and allows frequent clonal monitoring without repeated BM biopsies.

Several limitations of this study should be acknowledged. The extended inclusion period from 1982 to 2022 may have resulted in methodological heterogeneity. However, diagnostic procedures within MDS registry have been established in the mid‐1990s, including standardized cytomorphology, histology and standardized chromosomal analysis in the Institute of Human Genetics, University Hospital Düsseldorf. Cytogenetic analyses were performed using consistent laboratory protocols and were evaluated by an experienced cytogeneticist, thereby minimizing methodological variability over the study period. Furthermore, it should be noted that CCB from PB may have limited sensitivity for detecting low frequency subclonal abnormalities. Small cytogenetic subclones present in BM may remain undetected in PB due to their lower abundance. Nevertheless, the high concordance observed in the number of aberrant metaphases between PB and BM, together with a largely consistent cytogenetic risk stratification, indicates that this limitation did not substantially affect the overall conclusions of this study. Additionally, only 63 temporally matched PB and BM sample pairs were available for direct comparison, limiting statistical power and robustness of the concordance analyses. Finally, relevant clinical variables, including treatment status and comorbidities, were not systematically incorporated into the analysis. These factors may influence clonal dynamics and cytogenetic findings and therefore represent potential sources of bias. Both the limited paired cohort and the lack of comprehensive clinical data should be addressed in future prospective studies.

In summary, the present study suggests that, with optimized culture conditions, CCB from PB adequately reflects the clonal architecture observed in BM in a substantial proportion of MDS patients. Chromosomal analyses from PB demonstrate numbers of metaphases comparable to those observed in BM without systematic underrepresentation of aberrant clones and without clinically relevant differences in subsequent IPSS‐R or IPSS‐M risk stratification. Still, BM biopsies remain mandatory at initial diagnosis and in selected clinical scenarios, as the diagnosis generally requires morphological assessment and demonstration of dysplasia. However, PB‐based CCB and FISH represent a useful approach for routine follow‐up, drastically limiting the need for invasive BM testing.

## Funding

The authors have nothing to report.

## Conflicts of Interest

Sascha Dietrich: Speakers honorarium and scientific funding: Roche und Kite, BMS. Ulrich Germing: Speakers honorarium: BMS, Novartis, Janssen, Institutional research funding: BMS, Novartis, Abbvie, Jazz. The other authors declare no conflicts of interest.

## Data Availability

The data that support the findings of this study are available on request from the corresponding author. The data are not publicly available due to privacy or ethical restrictions.
